# DNA Methylation, Aging, and Cancer

**DOI:** 10.3390/epigenomes9020018

**Published:** 2025-06-03

**Authors:** Himani Vaidya, Jaroslav Jelinek, Jean-Pierre J. Issa

**Affiliations:** Coriell Institute for Medical Research, Camden, NJ 08103, USA; hvaidya@coriell.org (H.V.); jjelinek@coriell.org (J.J.)

**Keywords:** DNA methylation, aging, cancer

## Abstract

Aging and cancer, though distinct biological processes, share overlapping molecular pathways, particularly in epigenetic regulation. Among these, DNA methylation is central to mediating gene expression, maintaining cellular identity, and regulating genome stability. This review explores how age-associated changes in DNA methylation, characterized by both global hypomethylation and focal hypermethylation, contribute to the emergence of cancer. We discuss mechanisms of DNA methylation drift, the development of epigenetic clocks, and the role of entropy and epigenetic mosaicism, in aging and tumorigenesis. Emphasis is placed on how stochastic methylation errors accumulate in aging cells and lead to epiallelic shifts and gene silencing, predisposing tissues to malignant transformation, even despite recently increased cancer incidences at younger ages. We also highlight the translational potential of DNA methylation-based biomarkers, and therapeutic targets, in age-related diseases. By framing cancer as a disease of accelerated epigenetic aging, this review offers a unifying perspective and calls for age-aware approaches to both basic research and clinical oncology.

## 1. Introduction

Epigenetics, meaning “above genetics”, refers to mechanisms that supplement genetics in the inheritance of acquired phenotypes. The term was first introduced in 1942 by Conrad Waddington, who used this metaphor to describe how gene regulation controls development, regulates cellular plasticity, and adapts to environmental changes [1]. The current definition of epigenetics is the study of heritable phenotypic changes that result from modifications in gene expression, rather than alterations in the genetic code. These changes must be passed on and preserved through cell division. Epigenetic information dictates how cells transcribe the genome through chemical molecules in and around DNA, without changing the DNA itself. These molecules can be altered to switch genes on or off by changing chromatin structure into “closed” heterochromatin (repressed genes) or “open” euchromatin (expressed genes). The two most widely studied epigenetic modifications are DNA methylation and histone modification.

DNA methylation is an epigenetic modification where a methyl group from S-adenosyl methionine (SAM, derived from methionine) is added to the C5 position of cytosine, forming 5-methylcytosine (5mC). This typically occurs on cytosine bases followed by guanine, (i.e., CpG dinucleotides) [2]. 5mC accounts for only about 1% of nucleic acids in the human genome, as CpG sites are underrepresented due to the mutagenic potential of 5mC, which can spontaneously deaminate into thymine [3,4]. DNA methylation patterns are established by enzymes called DNA methyltransferases (DNMTs). DNA methylation patterns are first established de novo, during embryogenesis, by DNMT3A and DNMT3B [5]. DNMT1 then maintains DNA methylation during cellular replication by targeting the daughter strand of hemimethylated DNA [6]. TET enzymes (ten-eleven translocation methylcytosine dioxygenases) remove DNA methylation by oxidizing 5-methylcytosine (5mC) to 5-hydroxymethylcytosine (5hmC), which can further oxidize to 5-carboxylcytosine (5caC), which is excised by thymine DNA glycosylase (TDG). Base excision repair (BER) pathways then fill the gap, completing 5mC demethylation [7].

DNA methylation regulates gene expression based on CpG dinucleotide location. CpG islands (CGIs), genomic regions with a high CG to GC ratio, are usually unmethylated near promoters, keeping genes active. Methylation of these islands silences genes by Polycomb complexes, leading to heterochromatin. This silencing is common in inactive X-chromosomes and imprinted genes. Approximately 50% of gene promoters are within CGIs, especially highly conserved housekeeping genes. Methylation patterns are generally stable, maintaining epigenetic silencing, such as in X-inactivation and imprinting, through cell divisions.

## 2. DNA Methylation and Its Machinery

DNA methylation was first dubbed “epi-cytosine” by Hotchkiss in 1948 [8]. In the 1970s, 5-methylcytosine (5mC) was linked to gene regulation, leading to the discovery of its role in gene repression and CpG islands [9]. This epigenetic modification involves the transfer of a methyl group from S-adenosyl methionine (SAM) to the C5 position of cytosine [10].

DNA methyltransferases (DNMTs) are known as the “writers” of DNA methylation. During embryogenesis, following genome-wide erasure, de novo methylation sets a foundational methylation landscape, a task primarily carried out by DNMT3A and DNMT3B [5]. DNMT1, on the other hand, maintains these patterns by recognizing hemimethylated DNA and copying the methylation marks from the parent strand to the daughter strand during replication [6]. Other members of the DNMT family include DNMT3L, which lacks catalytic activity (due to the absence of a methyltransferase domain) and associates with DNMT3A and DNMT3B, helping their catalytic activities [11,12], and DNMT2, which primarily functions as an RNA methyltransferase [13].

DNA methylation-binding proteins (MBPs) are “readers” of DNA methylation marks and can repress transcription through recruitment of chromatin modification enzymes, gene silencing, and decreased transcription factor binding [14,15,16]. DNA methylation also attracts methyl-CpG-binding domain (MBD) proteins, some of which show a strong affinity for CpG sites and are closely associated with regions of increased methylation [17,18]. In particular, methyl-CpG-binding protein 2 (MeCP2) is an MBD that interacts with histone deacetylases (HDACs) and nucleosome-remodeling complexes, contributing to gene silencing [19,20]. One of these, UHRF1/2, recruits DNMT1 to hemimethylated DNA, and its overexpression plays a role in the silencing of tumor suppressor genes, as well as in migration, proliferation, and metastasis in various cancers [21,22].

Ten-eleven translocation (TET) enzymes act as a DNA methylation “eraser” by converting 5-methylcytosine (5mC) to 5-hydroxymethylcytosine (5hmC), which is further processed and removed via base excision repair (BER), resulting in DNA demethylation [7]. TET2 loss-of-function mutations can lead to defects in active DNA demethylation and various types of cancers [23,24,25]. Together, these molecular “writers”, “readers”, and “erasers” ensure precise control of gene expression, enabling cells to respond to developmental cues and environmental signals while maintaining genome stability and identity. However, dysregulation of any component in this network can lead to aberrant methylation patterns, contributing to developmental disorders, impaired cellular function, and diseases such as cancer, where tumor suppressor genes may become inappropriately silenced and/or oncogenes activated.

The precise regulation of DNA methylation is essential for maintaining normal cellular function and genomic stability. Disruption in the DNA methylation machinery, whether through mutations, altered expression, or dysregulated activity of key enzymes such as DNMTs, TETs, or methyl-binding proteins, can lead to aberrant epigenetic landscapes that contribute to oncogenesis. For instance, hypermethylation of promoter CpG islands in tumor suppressor genes can result in their transcriptional silencing, effectively removing critical brakes on cell proliferation and survival [26,27]. Conversely, global hypomethylation may activate oncogenes and promote chromosomal instability, further fueling tumor progression [28,29,30]. Mutations in TET enzymes, especially TET2, impair active DNA demethylation, causing accumulation of aberrant methylation marks and altered gene expression patterns that favor malignant transformation [23,31,32,33]. Overexpression of DNMT1 or recruitment of DNMTs by proteins such as UHRF1/2 can exacerbate hypermethylation-mediated silencing of genes that regulate cell cycle, apoptosis, and DNA repair, all of which are hallmarks of cancer [34,35]. Collectively, these disruptions in the methylation network contribute to the initiation, progression, and metastasis of various cancers by enabling tumor cells to evade growth control, resist apoptosis, and adapt to environmental pressures [36,37,38]. 

## 3. DNA Methylation and Cancer

While early studies of the association between DNA methylation levels and cancer produced mixed results, one pioneering study revealed significant hypomethylation of certain genes in cancer compared to normal tissues, with hypomethylation becoming even more pronounced during metastasis [39]. Another study identified conserved, low-methylation domains maintained by DNMT3A, containing both repressive and active histone marks, which may influence hematopoiesis and contribute to leukemia progression when dysregulated [40]. Additionally, the Polycomb complex has been shown to promote this hypomethylation [41]. Studies also show that DNMT1 disruption in mice causes hypomethylation and chromosomal instability, which can drive cancer development [42]. DNA hypomethylation can derepress imprinted genes and transposable elements, leading to disruption of cellular identity and cancer development [43,44,45]. Additionally, hypomethylation can enhance the expression of transposable elements, which may activate oncogenes and promote tumor formation [46]. Hypomethylation and expression of repetitive elements (e.g., LINE1) have been reported in multiple cancers and could be used as a biomarker [47]. A recent study demonstrated that the use of demethylating agents such as decitabine and azacytidine can induce the expression of the oncogene *SALL4* in patients with myelodysplastic syndrome (MDS), warranting caution in the use of these drugs. To further investigate, CRISPR-DNMT1-interacting RNA (CRISPR-DiR), a locus-specific demethylation technique, was employed to precisely target and hypomethylate a CpG island critical for *SALL4* expression. This resulted in similar hypomethylation, reinforcing the link between oncogene demethylation, re-expression, and tumorigenesis [48]. Another prostate cancer study found that androgen-response genes in tumor samples were hypomethylated compared to normal tissues [49]. Since prostate cancer and its treatment are driven by androgen receptors, this highlights the role of cancer-associated hypomethylation in the overexpression of oncogenic drivers.

Aberrant tumor landscape DNA methylation often presents as focal hypermethylation at CpG islands (CGIs) and global hypomethylation [50]. In cancer cells, hypermethylation is often found in transcriptional regulatory elements such as promoters and enhancers, including silencing of tumor suppressor genes (TSGs) in about 5–10% of CGIs [51]. Hypermethylation of CGIs in gene promoters can promote a repressive chromatin environment that obstructs transcription factor binding and inhibits gene transcription [10]. Aberrant promoter methylation, like mutations, can confer a selective advantage to neoplastic cells by silencing gene function (mostly TSGs). For example, genes such as *VHL*, *BRCA1*, *STK11*, *MLH1*, and *MGMT*, linked to familial renal, breast, and colon cancers, are frequently epigenetically silenced in their sporadic forms [52]. Other examples include the hypermethylation of promoter regions in genes that regulate cell cycle, apoptosis, cellular adhesion, and embryonic development, likewise linked to tumor formation in various cancers [26]. This DNA methylation-induced silencing of TSGs can recruit methyl-binding proteins and Polycomb repressive complexes to form heterochromatin [53]. For example, the earliest known example of promoter region hypermethylation of a TSG was that of *RB1* in patients with retinoblastoma [54].

Certain cancers exhibit nonrandom, widespread hypermethylation of CGIs in the promoters of multiple genes, a phenomenon known as the CpG island methylator phenotype (CIMP). CIMP was initially characterized by evaluating the methylation status of 30 different methylation-in-tumor (MINT) loci, and three well-known TSGs, in colorectal cancer (CRC) tumors and adenoma samples [55]. Although there is no standardized classification for specific methylated loci used to define CIMP, researchers commonly examine five key loci: *hMLH1*, *p16*, *MINT1*, *MINT2*, and *MINT31*. Additionally, other genes, such as *IGFBP3*, *NEUROG1*, *RUNX3*, *CRABP1*, *HIC1*, *SOCS1*, *CACNA1G*, *IGF2*, and *WRN*, are also evaluated [56]. CIMP has also been described in other cancers such as glioblastoma, gastric cancer, prostate, and hepatocellular, etc. [57]. Loss of *TET* genes, through mutations, results in hypermethylation of enhancers, which, combined with other oncogenic mutations, can contribute to malignant transformation of cancer cells in patients with clonal hematopoiesis of indeterminate potential (CHIP), clonal cytopenia of undetermined significance (CCUS), and acute myeloid leukemia (AML) [58].

The initiation and progression of tumorigenesis can be driven by a combination of genetic, epigenetic, and environmental factors. Genetic mutations affecting epigenetic modifiers, such as chromatin-remodeling complexes and DNA methylation regulators (“writers”, “readers”, and “erasers”), play a significant role in cancer onset and prognosis. These epigenetic alterations not only serve as biomarkers and therapeutic targets but also suggest that epigenomic dysregulation plays a causal role in cancer development and metastatic progression [59]. A recent study in Drosophila demonstrated that genetic mutations are not essential for cancer onset. The researchers used RNA interference (RNAi) to induce a transient loss of the Polycomb group (PcG) protein PH, a component of Polycomb repressive complex 1 (PRC1), by knocking down both PH isoforms in the developing Drosophila larval eye imaginal disc. A brief (24-h) loss of PH, during the larval stage, was sufficient to irreversibly induce tumor formation, driven by derepression of the JAK-STAT pathway and the *zfh1* oncogene, without the presence of driver mutations. This finding suggests that cancer can arise purely from epigenetic dysregulation, offering valuable insights and potential therapeutic strategies for cancers with low mutational loads [60].

Although cancer genomes often exhibit global hypomethylation, this occurs, paradoxically, alongside the frequent upregulation of DNA methyltransferase 1 (DNMT1) and its cofactor, UHRF1, in many cancers [61,62]. This contradiction could be explained through the differential targeting of methylation within the genome. Despite global hypomethylation (largely in repetitive elements), focal hypermethylation occurs at specific gene promoters, especially tumor suppressor genes [27]. Although the primary role of DNA methylation writers, readers, and erasers is to maintain proper methylation patterns, their over- or under-expression can disrupt this balance, contributing to aberrant methylation of normally unmethylated gene promoters (e.g., in tumor suppressor genes), and/or hypomethylation and activation of oncogenes, disrupting global methylation fidelity. One possible mechanism related to DNMT1/UHRF1 upregulation, leading to cancers, could be disturbed DNMT1 function due to post-translational modifications, leading to aberrant methylation activity [34,35]. Likewise, UHRF1 overexpression could also drive mislocalization of DNMT1, leading to hypomethylation [63]. While the conflicting phenomena of global hypomethylation and focal hypermethylation could be based merely on correlative analyses, studies consistently reveal associations between methylation patterns and disease states; however, this does not necessarily demonstrate causality. Emerging experimental approaches, such as CRISPR/dCas9-based epigenetic editing, are beginning to clarify causal relationships, although such investigations remain limited and ongoing [64].

## 4. DNA Methylation and Aging

The first study showing that global loss of DNA methylation is inversely related to lifespan was in rodents [65]. One of the earliest genes studied in relation to age-associated DNA methylation was estrogen receptor-alpha (ERα), where methylation levels in the human colon can increase by 1% every three years [66], similar to genes such as *N33* and *MYOD* [67]. Another genome-wide study revealed that approximately 50% of promoters that gain methylation with age are the same as those that gain methylation during the pathogenesis of colon cancer [56], suggesting that promoters that gain methylation during cancer development also undergo increased methylation with age. Most CGIs remain unmethylated in normal tissues, except for a few involved in tissue differentiation [68]. A study of autopsy samples found that, in non-neoplastic tissues, methylated promoters become more frequent in individuals over 42 years, mirroring an increased rate of malignancy, with significant tissue variability [69]. This indicates that methylation changes in promoter-CGIs may be a pathological event associated with aging.

Age-related DNA methylation change is bidirectional: while some CpG sites gain methylation, others lose it. These changes are not random; CpG islands typically gain methylation, while repeat regions such as SINEs and LINEs tend to lose it [70]. This cannot be attributed solely to enzymatic changes in DNMT or TET levels. Age-related methylation changes vary with tissue type and are conserved across species, with highly proliferative tissues experiencing the most pronounced alterations with age [71]. Studies have shown that mutations in epigenetic regulators such as TET2, IDH1, and DNMT3A, as well as altered expression of DNMT3A and DNMT3B, can drive age-related changes in DNA methylation [72]. Other factors contributing to age-related DNA methylation changes include altered enzymatic activity and metabolic processes, e.g., acetyl coenzyme A (acetyl-CoA), SAM, and their coenzymes [73]. Additionally, TET activity can cause passive demethylation, and spontaneous deamination of cytosine to uracil can further alter methylation [74,75].

Another cause of change in DNA methylation with age could be loss of SET8, a cell cycle-regulated protein methyltransferase that prevents UHRF1 ubiquitination and degradation, with aberrant recruitment of DNMT1 by UHRF1 facilitating histone H3 ubiquitination at lys18/23, leading to increased DNA methylation [76]. Differentially methylated regions (DMRs), hypermethylated with age, have been associated with Polycomb repressive complex 2 (PRC2), facilitating heterochromatin formation by recruiting different enzymes that promote DNA methylation and repressive histone methylation marks [77]. Other studies have shown that CpG-dense regions expand methylation to neighboring CpG sites, and that solo CpG sites are more susceptible to DNA methylation loss [78].

DNA hypomethylation with age can also result from DNMT1 errors during cell division. As DNA replicates, strands become hemimethylated, and new strands are methylated by DNMT1, following the methylation pattern from the parent strand; however, DNMT1 is not foolproof and can allow errors [79], leading to hypomethylation. Another study also revealed that DNMT1 activity decreases with aging, contributing to global DNA hypomethylation [80]. Enhanced replication can also lead to increased DNA methylation. A study using hairpin DNA sequencing found that daughter strands can gain up to 5% more methylation compared to parent strands [81]. DNA methylation errors accumulate due to the lack of strict checkpoints, and even treatment with demethylating agents does not prevent surviving cells from re-entering the cell cycle and dividing [82].

## 5. DNA Methylation Drift, Correlation with Lifespan, and Epigenetic Clocks

DNA methylation drift can be seen as the gradual erosion of established methylation patterns with age, characterized by both gains and losses of methylation at different genomic locations over the lifespan of a species. Studies have shown that DNA methylation drift with age also correlates with the lifespan of different species, such as whales, dogs, mice, monkeys, and humans, allowing correlative studies across multiple species [83,84]. One study, which focused on genes hypermethylated with age and conserved across three species, mice, monkeys, and humans, found that species with a longer lifespan have slower methylation drift rates [84]. Quantification of the drift rate can serve as a biomarker to study and diagnose age-related diseases. Similar studies, done in different bat species with different lifespans, showed similar results where lifespan correlated with DNA methylation drift, while a study done in naked mole rats showed that the queen mole rat had a slower drift [85,86]. Together, these studies support the mitotic clock hypothesis that proposes that age-related DNA methylation errors arise primarily from errors in methylation maintenance during stem cell division. These errors are propagated to daughter cells over time, contributing to cellular dysfunction and ultimately enabling the development of epigenetic clocks.

The first epigenetic clock identified 71 CpG sites that could predict chronological age and detect accelerated methylation outliers [87]. Horvath later developed a pan-tissue clock, using 353 sites, to predict age across various tissues [88]. While these clocks successfully estimate chronological age, predicting biological age is crucial for assessing the prognosis of age-related diseases. Since individuals of the same chronological age can age differently biologically, these clocks could identify those experiencing accelerated aging, allowing for earlier screenings, preventive measures, and lifestyle changes to delay disease onset. Researchers have used the Hannum and Horvath clocks as foundational models to develop many additional epigenetic clocks that incorporate factors such as smoking, environmental exposures, and lifestyle. These advanced clocks aim to predict age reversal, mortality, cellular reprogramming, biological aging, and disease risk. Non-human aging clocks for animal models, such as rodents, dogs, whales, etc., have also been developed [89]. DNA methylation changes happen as early as embryogenesis and continue as the organism grows older; in humans, however, there appears to be a survivor’s bias where more older people are biologically younger [90]. These findings underscore the need for more comprehensive studies of age-related DNA methylation changes to develop tools for measuring biological age, which could be instrumental in preventing age-related diseases.

## 6. Epigenetic Mosaicism and Epialleles

Although cells within tissues and organs share the same genomic DNA, they perform different functions and express different genes. Even cells from homogeneous or clonal populations display cell-to-cell heterogeneity [91,92]. Single-cell analysis has revealed phenotypic heterogeneity in clonal populations that affects transcription factors and cellular signaling [93]. Even human induced pluripotent cells (iPSCs) have different subpopulations of cells with different cell state transitions [94]. A key source of cellular heterogeneity is epigenetic mosaicism. As previously mentioned, DNA methylation errors gradually accumulate, leading to disrupted gene regulation. These errors are not isolated to individual cells; instead, they are faithfully inherited by daughter cells during cell division. This propagation of methylation errors across cellular generations contributes to the gradual decline in cellular function and the onset of age-related conditions. These age-related heterogeneous changes can only be attributed to the process of stochastic methylation drift, where random fluctuations in DNA methylation patterns accumulate over time, leading to variability in gene expression and cellular function.

Further support of this theory can be garnered by studies of identical twins that, despite sharing the same genome and being raised in identical environments, develop distinct methylation profiles as they age [95]. In an early investigation into epigenetic mosaicism and the stochastic development of differentially methylated regions, researchers monitored the in vitro evolution of immortalized fibroblasts across 300 generations, indicating that methylation changes were localized and uncorrelated, consistent with a random, stochastic process [96]. Next-generation sequencing methods have also shown DMRs at base-pair resolution in cancerous, normal, and developmental cells [97]. One potential cause of epigenetic mosaicism is the accumulation of methylation errors in stem cells. As stem cells asymmetrically divide, whether to replenish the stem cell pool or differentiate into specialized cells, these errors may accumulate, contributing to the mosaicism observed. Research indicates that higher mitotic age is associated with an increase in aberrant epigenetic modifications and mutations [98]. In cancerous and adjacent tissues, cell division rates are linked to abnormal methylation [99]. Studies of stem cells from tissues like skeletal muscle, intestine, hematopoietic, and germline show that DNA methylation polymorphisms rise with age [100,101,102]. Single-cell analysis of liver cells also revealed that epigenetic polymorphisms outnumber somatic mutations and increase with age [103]. Epialleles refer to specific DNA methylation patterns at a genetic locus, where all CpG sites in a single sequencing read form an epigenetic haplotype. These methylation patterns, and the rate at which they change, can be instrumental in studying epigenetic mosaicism. In younger cells or organisms, epialleles may exhibit uniformity, but as DNA methylation undergoes alterations with age, an epiallelic shift may occur. Measuring the frequency of this shift could provide important insights into epigenetic mosaicism, DNA methylation drift with age, and age-related diseases.

Current attempts in quantifying epigenetic mosaicism rely on information theory to measure disorder in the system. Cellular variability, in a mostly homogenous population, can be caused by small changes in epialleles, possibly detectable by proposed methods. One method to quantify epigenetic mosaicism is to use the principles of entropy, defined as a measure of randomness or disorder of a system. The second law of thermodynamics states that entropy can only increase with time in an isolated or closed system. Biologically, entropy can be quantified as degradation or change in RNA, DNA, or protein structures over the lifespan. The concept of using entropy to measure a biological phenomenon is not new and was proposed in the 1970s [104], with advances in next-generation sequencing now revealing the polymorphic and stochastic nature of DNA methylation. The age-related gain or loss of methylation mirrors the increase in randomness or disorder associated with rising entropy. For example, young cells typically exhibit a uniformly methylated or unmethylated state, reflecting low entropy. As DNA methylation drifts with age, it transitions into a more disordered state, indicative of high entropy. Studies have quantified this change in entropy with age using different methods, including Shannon’s entropy, a concept borrowed from information theory to measure the uncertainty of the occurrence of a certain event; this was used by Lou et al. to measure brain entropy [105]. Researchers suggest using combinatorial entropy, relative entropy, and statistical entropy to study other aging biological functions such as chronic inflammation, aging epidermis, and arrhythmia [106]. Another study quantified the epigenetic changes in a pair of twins, where one twin stayed in the International Space Station for a long duration, to measure the effect of space and low gravity on DNA methylation, with methylation stochastics measured by genome-wide mean methylation levels (MMLs) and normalized methylation entropy (NME) [107]. NME measures the randomness or disorder of methylation patterns at a given locus by assessing the distribution of methylation states across cell populations. It is calculated by first determining the Shannon entropy of methylation states at CpG sites and normalizing this against the maximum possible entropy for that context, providing a standardized metric for methylation variability across the genome [108]. Other scientists have proposed combining entropy and probability distributions to measure DNA methylation entropy, a method known as the Jensen–Shannon distance (JSD) [108]. Specifically, JSD measures the divergence between two probability distributions, such as an observed methylation pattern versus a baseline or standard pattern, making it a useful tool for comparing methylation profiles across samples, via capturing both variability and distributional differences [108]. Vaidya et al. (2023) used JSD to show that DNA methylation entropy increases with age; however, this increase is dependent upon cellular replication, with cells with faster turnover rates (e.g., the intestinal epithelium) having more changes in entropy compared to cells from other organs (e.g., heart), with less cellular division [109].

There are other approaches to quantify the difference in epialleles with age or disease. One such method is cell heterogeneity-adjusted clonal methylation (CHALM). While this method uses standard percent methylation, it considers a location methylated if any CpG site on a read is methylated. Thus, this method treats even minimal local methylation as equivalent to full methylation of the region, and more strongly correlates with gene expression. This is especially evident in lowly methylated promoters, indicating that even a small degree of methylation in regulatory regions can significantly disrupt gene expression [110]. Another approach is to isolate heterogeneous epialleles by a digital high-resolution melt to profile methylation patterns. O’Keeffe et.al. developed this method to find that biomarkers in ovarian cancer cells exhibit heterogeneous methylation patterns [111]. There are caveats to using entropy as a measure of biological change, and it can be confounded by cellular heterogeneity or imprinted genes. Thus, this needs to be used in conjunction with other methods such as percent methylation.

## 7. Blurring the Epigenetic Line Between Youth and Age in Cancer Development

Recent advancements in single-cell epigenomic technologies have unveiled substantial heterogeneity in chromatin states across both cancerous and normal tissues. The development of metrics such as epiCHAOS has enabled the quantification of cell-to-cell epigenetic variability, revealing associations with stemness and plasticity in various biological systems, including malignancies [112]. Such heterogeneity is not confined to aged cells; environmental exposures (e.g., pollutants), inflammation, and lifestyle factors can induce epigenetic aberrations even in younger individuals, potentially accelerating epigenetic aging and increasing cancer susceptibility [113]. This perspective aligns with the Developmental Origins of Health and Disease (DOHaD) hypothesis, which posits that early-life environmental exposures can reprogram the epigenome, thereby influencing disease risk later in life [114]. Consequently, framing epigenetic patterns strictly as “young” or “aged” may oversimplify the inherently dynamic and context-dependent nature of the epigenome, highlighting the importance of a more nuanced understanding of how environmental exposures shape epigenetic heterogeneity and influence cancer risk.

The recent global rise in early-onset cancers, particularly colorectal cancer (CRC), has prompted significant concern and investigation. Epidemiological studies report a consistent increase in CRC incidence among individuals under 50 years of age, in multiple countries [115], a trend that contrasts with declining rates in older populations, due to screening and prevention efforts [116]. While the precise drivers remain unclear, mounting evidence implicates early-life exposures to environmental and dietary risk factors such as high intake of processed foods, sedentary behavior, antibiotic use, obesity, and microbiome disruption as contributors to this shift [117]. Importantly, single-cell epigenomic analyses of colorectal tissues demonstrate the occurrence of epigenetic reprogramming and chromatin accessibility changes, even at premalignant stages and absent canonical genetic drivers [118]. For example, chromatin variants in precancerous colonic cells can promote stem-like phenotypes that potentially prime tissues for malignancy. When such epigenetic alterations co-occur with early-life somatic mutations introduced by DNA-damaging agents, they may synergistically accelerate tumorigenesis [119]. This interplay underscores a need to re-evaluate current risk models and screening strategies for younger populations, incorporating molecular markers of early epigenetic aging and inflammation-related chromatin remodeling as predictive tools.

## 8. Aging, Cancer, and DNA Methylation: A Discussion

Despite its increasing incidence in younger populations (previous section), cancer remains, predominantly, a disease of age. The National Cancer Institute (NCI) lists age as the primary risk factor for cancer, influencing overall cancer risk and many specific cancer types. Cancer incidence rates rise steadily with age, starting from under 25 cases per 100,000 individuals in those younger than 20, increasing to around 350 cases per 100,000 individuals aged 45 to 49, and surpassing 1000 cases per 100,000 individuals aged 60 and older. Recognizing cancer as an age-related disease is crucial, necessitating consideration of age in both benchwork and clinical trials. Regrettably, mouse studies usually involve those aged 4–6 weeks (equivalent to 15–20 human years), and clinical trials often lack representation from individuals over 75 [120]. Such limitations may preclude ideal conditions to comprehend the full complexity of cancer, diminishing the improvement of therapeutic approaches. Overall, cancer can be viewed as a form of accelerated aging, with more than 70% of aberrant DNA methylation changes in cancer occurring during aging [55].

**Figure 1:**
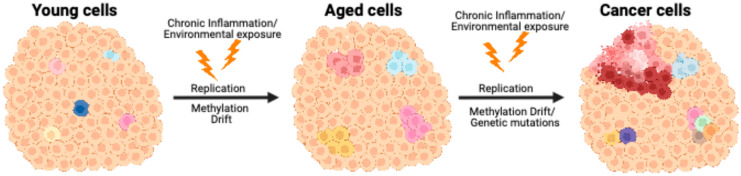
Model of how age-related DNA methylation changes contribute to focal diseases, specifically cancer. In young normal tissues (**left**), most cells exhibit uniform epigenetic patterns, with occasional outliers showing subtle variation related to genetics or the environment. Age-related DNA methylation changes result in epigenetic mosaicism (**middle**), as represented by the differently colored cells, showing cell-to-cell epigenetic variation. Further epigenetic changes, combined with DNA mutations or carcinogen exposure, transform the cells into a full-fledged malignancy (**right**).

As previously mentioned, cancer cells accumulate aberrant DNA methylation, as well as other epigenetic alterations. Some of these methylation changes occur in promoter CpG islands of tumor suppressor genes, downregulating their expression, while others occur in transposable elements, whose increased levels can promote oncogene expression [43], through genomic instability, as these elements can insert into various genomic regions, potentially disrupting gene function and regulatory regions [121]. Hypomethylation of specific transposable elements, such as LINE-1 sequences, could also activate alternative promoters within oncogenes. In bladder cancer, for example, loss of methylation at a LINE-1 promoter has been shown to initiate an alternative transcript of the MET oncogene, thereby promoting tumorigenesis [122]. These DNA methylation changes, when combined with genetic defects, could provide cancer cells with an advantage in cellular proliferation and drug resistance. Also, a recent study showed that purely epigenetic changes can cause tumorigenesis in Drosophila [60], emphasizing how epigenetic changes initiate cancer. Other studies have also shown that mutations in DNA methylation machinery, such as DNMTs, TETs, and UHRF1, can also cause neoplasia. DNA methylation changes not only occur with age but also with environmental changes; these changes, combined with genetic hits, can concoct a “perfect storm” that leads to neoplastic lesions (Figure 1). Thus, it is advantageous to study DNA methylation change as a function of age that leads to cancer, using various methods to quantify those changes, such as percent methylation, epialleles (epigenetic mosaicism), and entropy. These approaches provide valuable insights into how aging-associated alterations in methylation patterns may contribute to carcinogenesis and help in the diagnosis, treatment, and prevention of age-related diseases.

## 9. Conclusions and Future Perspectives

The interplay between aging, DNA methylation, and cancer represents a critical axis in understanding tumorigenesis. As discussed, epigenetic alterations, particularly age-associated promoter hypermethylation of tumor suppressor genes and hypomethylation of transposable elements, contribute significantly to oncogenic transformation. These changes, especially when occurring alongside genetic mutations, support a model in which cancer can be viewed as an epigenetic consequence of aging. Despite these advances, current research frameworks often fall short in capturing the complexity of age-related cancer biology. Preclinical studies remain heavily reliant on young animal models, and clinical trials frequently underrepresent older adults, leading to gaps in translational relevance and therapeutic development. Bridging this disconnect requires a shift toward age-inclusive study designs that more accurately reflect the demographics of cancer incidence.

Recent technological developments offer promising new avenues for addressing these limitations. Single-cell methylome analysis now allows for high-resolution interrogation of epigenetic heterogeneity, capturing dynamic and cell-type-specific changes that bulk analyses obscure. These insights are particularly valuable in the context of aging, where epigenetic drift and mosaicism play key roles in disease onset. Furthermore, CRISPR-Cas-based epigenome editing, especially tools targeting DNA methylation regulators such as DNMTs and TET enzymes, enables precise manipulation of epigenetic marks at specific loci. These technologies not only serve as powerful research tools to establish causal links between methylation and gene function but also hold therapeutic potential in correcting pathogenic epigenetic alterations. Future research should aim to integrate these approaches into longitudinal studies that track epigenetic changes over the lifespan. Such efforts will be essential in identifying early biomarkers of cancer risk, refining our understanding of tumor evolution in aging tissues, and developing targeted epigenetic therapies. As the field continues to evolve, leveraging emerging technologies in the context of aging will be crucial to improving cancer prevention, detection, and treatment.
